# Performance of SARS-CoV-2 Antigens in a Multiplex Bead Assay for Integrated Serological Surveillance of Neglected Tropical and Other Diseases

**DOI:** 10.4269/ajtmh.22-0078

**Published:** 2022-06-27

**Authors:** Sarah Gwyn, Ado Abubakar, Oluwaseun Akinmulero, Eric Bergeron, Ugboaja Nkechi Blessing, Jasmine Chaitram, Melissa M. Coughlin, Ayuba B. Dawurung, Felicia Nwatu Dickson, Mudiaga Esiekpe, Erasogie Evbuomwan, Stacie M. Greby, Nnaemeka C. Iriemenam, Markus H. Kainulainen, Thomas Andrew Naanpoen, Loveth Napoloen, Ifeanyichukwu Odoh, McPaul Okoye, Temitope Olaleye, Amy J. Schuh, S. Michele Owen, Awala Samuel, Diana L. Martin

**Affiliations:** ^1^ Division of Parasitic Diseases and Malaria, Centers for Disease Control and Prevention, Atlanta, Georgia;; ^2^ Institute of Human Virology, Abuja, Nigeria;; ^3^ Division of High-Consequence Pathogens and Pathology, Centers for Disease Control and Prevention, Atlanta, Georgia;; ^4^ Division of Laboratory Systems, Centers for Disease Control and Prevention, Atlanta, Georgia;; ^5^ Division of Viral Diseases, Centers for Disease Control and Prevention, Atlanta, Georgia;; ^6^ Nigeria Centre for Disease Control, Abuja, Nigeria;; ^7^ Division of Global HIV and TB, Centers for Disease Control and Prevention, Abuja, Nigeria;; ^8^ National Center for HIV/AIDS, Viral Hepatitis, STD and TB Prevention, Atlanta, Georgia

## Abstract

Serosurveillance can provide estimates of population-level exposure to infectious pathogens and has been used extensively during the COVID-19 pandemic. Simultaneous, serological testing for multiple pathogens can be done using bead-based immunoassays to add value to disease-specific serosurveys. We conducted a validation of four SARS-CoV-2 antigens—full-length spike protein, two receptor binding domain proteins, and the nucleocapsid protein—on our existing multiplex bead assay (MBA) for enteric diseases, malaria, and vaccine preventable diseases. After determining the optimal conditions for coupling the antigens to microsphere beads, the sensitivity and specificity of the assay were determined on two instruments (Luminex-200 and MAGPIX) when testing singly (monoplex) versus combined (multiplex). Sensitivity was assessed using plasma from 87 real-time reverse transcription polymerase chain reaction (rRT-PCR) positive persons collected in March–May of 2020 and ranged from 94.3% to 96.6% for the different testing conditions. Specificity was assessed using 98 plasma specimens collected prior to December 2019 and plasma from 19 rRT-PCR negative persons and ranged from 97.4% to 100%. The positive percent agreement was 93.8% to 97.9% using 48 specimens collected > 21 days post-symptom onset, while the negative percent agreement was ≥ 99% for all antigens. Test performance was similar using monoplex or multiplex testing. Integrating SARS-CoV-2 serology with other diseases of public health interest could add significant value to public health programs that have suffered severe programmatic setbacks during the COVID-19 pandemic.

## INTRODUCTION

Severe acute respiratory syndrome coronavirus 2 (SARS-CoV-2), the virus that causes COVID-19, is a novel coronavirus (sarbecovirus) that emerged at the end of 2019. Coronavirus disease 2019 was declared a pandemic in March of 2020 and confirmed cases have now been reported in almost every country. Antibodies to SARS-CoV-2 antigens develop 1–3 weeks after infection.^1^^,^^2^ While of limited utility to diagnose an acute infection, antibodies can be used to estimate the proportion of a population with evidence of previous infection with SARS-CoV-2. Severe acute respiratory syndrome coronavirus 2 serosurveys have already been undertaken in places such as the United States,^3^^–^^5^ Peru,^6^ Kenya,^7^^,^^8^ Nigeria,^9^ United Arab Emirates,^10^ and Slovenia.^11^ A meta-analysis of 82 serosurveys conducted through December 22, 2020, 38 of which focused on the general population and often used convenience specimens, showed an average 8% antibody seroprevalence for the general population.^12^ However, North America and Europe were overrepresented in this analysis, and additional data are needed to understand the global burden of SARS-CoV-2 exposure.

Most commercially available SARS-CoV-2 serological assays detect antibodies to a single antigenic target, typically either the nucleocapsid (N) protein, full-length spike (S) protein, or the S receptor binding domain (RBD) proteins. However, serological assays based on a single antigenic target may not be ideal, especially in low prevalence settings or in differentiating natural infection from vaccination, when compared with a multi-antigen target.^13^ Additionally, single antigenic target serological assays may be affected by cross reactions with other endemic infections.^14^ Multiplex bead assays (MBA) used for integrated serological surveillance could add value to COVID-19 serosurveys by simultaneously testing for other infections of public health interest.^15^^,^^16^ We have used MBA for integrated serosurveillance of neglected tropical diseases (NTDs),^16^^–^^19^ malaria,^20^^–^^22^ waterborne diseases,^23^^,^^24^ and measuring seroprotection to vaccine preventable diseases (VPDs)^25^ to obtain additional information on disease burden, susceptibility to vaccine-preventable diseases, and progress toward disease elimination targets.

The customizable nature of multiplex platforms, such as Luminex-based technology, allows the addition of specific targets for new diseases to currently used panels in the assay. In this study, we describe the validation of four SARS-CoV-2 antigens using our existing MBA. We also assess the reproducibility of the assay between laboratories in two countries.

## METHODS

### Ethics.

This study did not involve contact with patients, and no specimens were collected specifically for the purposes of this study. Residual samples were used for diagnostics development under a protocol that was reviewed and approved by the Centers for Disease Control and Prevention Institutional Review Board with an approved waiver of informed consent (see 45 C.F.R. part 46; 21 C.F.R. part 56). The approved Project ID number is 0900f3eb81c1b13d. All methods involving human biological samples were carried out in accordance with relevant guidelines and regulation.

### Specimens.

Two panels of plasma specimens were used to determine the optimal conditions for coupling the SARS-CoV-2 antigens to beads: Panel 1 was comprised of seven SARS-CoV-2 antibody positive plasma specimens collected from patients confirmed positive by rRT-PCR or who had clinical signs/symptoms consistent with COVID-19 and Panel 2 was comprised of 11 plasma specimens obtained from healthy U.S. donors prior to 2019. Panel 3 (*N* = 204) was used to determine cutoff values and assay sensitivity and specificity and consisted of plasma collected from 106 individuals that had SARS-CoV-2 rRT-PCR results available (87 rRT-PCR positives and 19 rRT-PCR negative) during March–May of 2020, and 98 plasma specimens collected prior to December of 2019. Panels 4 and 5 were used to further evaluate assay performance. Panel 4 consisted of plasma from 108 rRT-PCR confirmed positive persons, 102 of which had information on the duration of time between COVID-19 symptom onset and blood collection. Among the latter, specimens were collected between 13 and 83 days post-symptom onset, with 54 collected ≤ 21 days post-symptom onset and 48 collected > 21 days post-symptom onset. Panel 5 included 86 specimens collected prior to 2019. Additional information on each panel is shown in Table 1. All plasma specimens were stored at −80°C prior to testing.

**Table 1:** Characteristics of plasma sample cohorts

Panel	Sample cohort	Country	Date of sample collection	*N*	Testing location
1	Suspected COVID-19 positive patients	The United States	April–May 2020	7	The United States
2	Healthy donors	The United States	2011	11	The United States and Nigeria
3	Healthy donors	The United States	October 2018–November 2019	98	The United States
3	Suspected COVID-19 positive patients	The United States	March–June 2020	106	The United States
4	COVID-19 confirmed positive patients	The United States	April–July 2020	108	The United States and Nigeria
5	Healthy donors	The United States	Prior to 2019	86	The United States

Suspected COVID-19 positive patient cohorts included donors that tested positive for SARS-CoV-2 by rRT-PCR (real-time reverse transcription polymerase chain reaction) and donors that had clinical signs/symptoms consistent with COVID-19 and household members testing SARS-CoV-2 positive. Healthy donor cohorts included samples collected prior to the beginning of the COVID-19 pandemic.

Artificial dried blood spots (DBS) were prepared by spiking red blood cells (RBCs) from an antibody-negative donor with equal parts plasma from panels 1 and 2 using previously described methods.^26^ Blood (10 µL) spiked with each specimen (*N* = 18) was transferred onto one filter paper extension (TropBio Pty Ltd., Townsville, Queensland, Australia) and dried overnight at 20–22°C. DBS were stored at −20°C prior to testing.

Six specimens for precision testing were selected from panels 1, 2, and 3 to ensure that high, medium, and low values in the linear range of the assay for each antigen were assessed.

### Antigens.

To allow secretion and affinity purification of SARS-CoV spike RBD (GenBank MN908947), residues 319–541 and 319–591 were fused to the C-terminal of IL-6 signal peptide, 8xHis tag, a glycine linker (GGGGS) and human rhinovirus 3C protease cleavage site. The human codon-optimized tagged RBD sequences were cloned into mammalian expression vector pEEV-Puro.^27^ Plasmid to produce the prefusion stabilized HexaPro spike^28^ trimer was kindly provided by Jason McLellan (University of Texas, Austin). Expi293 cells were grown in Expi293 expression medium (Thermo Fisher Scientific, Waltham, MA). The spike and His-RBD fusion proteins were produced by transfecting Expi293 cells using FectoPro reagent (Polyplus Transfection) and the secreted proteins were purified by immobilized metal affinity chromatography using HisTrap Excel columns (Cytiva) and dialyzed against PBS pH 7.4. SARS-CoV-2 nucleocapsid antigen was purchased commercially (GenScript, Piscataway, NJ, Cat # Z03480).

### Coupling antigen to microspheres.

Antigens were coupled to carboxylated MagPlex^®^ microspheres (Luminex Corporation, Austin, TX) at five concentrations (6, 3, 1.5, 0.6, and 0.3 µg of antigen per 1.25 × 10^6^ beads) in two coupling buffers (1× phosphate buffered saline [PBS] pH 7.2 and 50 mM MES, 0.85% NaCl at pH 5) following previously described methods.^24^

### MBA.

Sera and DBS were diluted to 1:400 in Buffer B (1× PBS, 0.5% casein, 0.5% polyvinyl alcohol [PVA], 0.8% polyvinylpyrrolidone [PVP], 0.3% Tween-20, 0.02% sodium azide, and 3 µg/mL *Escherichia coli* extract). Diluted specimens were tested in duplicate on an MBA as previously described and read on either a Bio-Plex 200 (BioRad, Hercules, CA) or MAGPIX (Luminex, Austin, TX) instrument (Priest and Moss). Briefly, diluted specimen (50 µL) was incubated with beads (1,250 beads/well/antigen) in either a 96-well filter bottom (Bio-Plex assay) or flat bottom (MAGPIX assay) plate for 1.5 hours. Beads were washed three times with 1× PBS + 0.05% Tween-20 (PBST). Beads in filter bottom plates were washed with 100 µL of PBST using vacuum filtration to remove liquid between each wash. Beads in flat bottom plates were washed with 200 µL PBST using a handheld manual magnetic plate washer (BioRad) to hold the beads to the plate while liquid was decanted. Beads were incubated with 50 ng IgG (Southern Biotech, Birmingham, AL) and 20 ng IgG4 (Southern Biotech) to detect bound antibody for 45 minutes. Beads were washed three times with PBST and incubated with 250 ng SAPE (Invitrogen, San Francisco, CA) for 30 minutes. Beads were washed three times with PBST and incubated with 1× PBS, 0.5% BSA, 0.05% Tween-20, and 0.02% NaN3 for 30 minutes to remove any loosely bound antibodies. Beads were washed one more time, suspended in 1× PBS and stored overnight at 4°C. The next day, beads were read on either the Bio-Plex 200 or the MAGPIX. The average median fluorescence intensity (MFI) with background (Buffer B alone) subtracted (MFI-bg) was determined for each antigen for each specimen.

Specimens were tested on both Bio-Plex and MAGPIX at US CDC in Atlanta, GA, and MAGPIX at the National Reference Laboratory (NRL) in Gaduwa, Nigeria. The location where each specimen set was run is shown in Table 1.

A 10-point positive pool (derived from panel 1) dilution series ranging from 1:100 to 1:51,200 was tested in monoplex (each coupling individually) and multiplex (all couplings combined). All specimens from panel 3 were tested in monoplex and multiplex. All other panels were tested only in multiplex.

Specimens for precision testing were run in duplicate on six plates on three different days (two plates/day)^29^ with multiple operators on both Bio-Plex and MAGPIX at CDC and on the MAGPIX at NRL.

### Statistical analysis.

Signal to noise ratios for each coupling condition were determined by dividing the average MFI of panel 1 by the average MFI of panel 2. The coupling condition that yielded the highest signal to noise ratio for each antigen was chosen as the optimal condition.

Linear regression analysis was performed to determine the coefficient of determination (R^2^) between results for each antigen derived from monoplex versus multiplex testing, from plasma versus DBSs, and from US CDC versus Nigeria NRL.

Cutoff values for each antigen were determined by receiver operating characteristic (ROC) curves using positive and negative specimens from panel 3, maximizing for sensitivity and specificity using Youden’s J index. Positives included plasma from 87 rRT-PCR positive persons and negatives included 98 pre-pandemic specimens and plasma from 19 rRT-PCR confirmed negative persons. Median fluorescence intensity-bg cutoff values for each antigen were chosen that maximized sensitivity and specificity using Youden’s J index. Sensitivity was determined as the percent of samples above the cutoff value out of the 87 positive samples. Specificity was determined as the percent of samples below the cutoff value out of the 117 negative samples. Area under the curve values was also determined. Analysis was performed using GraphPad Prism software.

Precision was determined by calculating the percent coefficient of variation (%CV) between plates for each specimen. Repeatability was determined using six plates run by one operator at U.S. CDC. Intermediate precision was determined using 12 plates run by two operators (six plates per operator) at U.S. CDC. Reproducibility was determined using 12 plates run at U.S. CDC and Nigeria NRL (six plates/site).

For additional assay evaluation, positive percent agreement (PPA) to rRT-PCR positive specimens was determined as the percent antibody positive out of all 108 specimens from panel 4. Negative percent agreement (NPA) was determined as the percent antibody negative out of 97 specimens from panels 2 and 5 (U.S. analysis).

## RESULTS

### Assay optimization.

**Figure 1.**
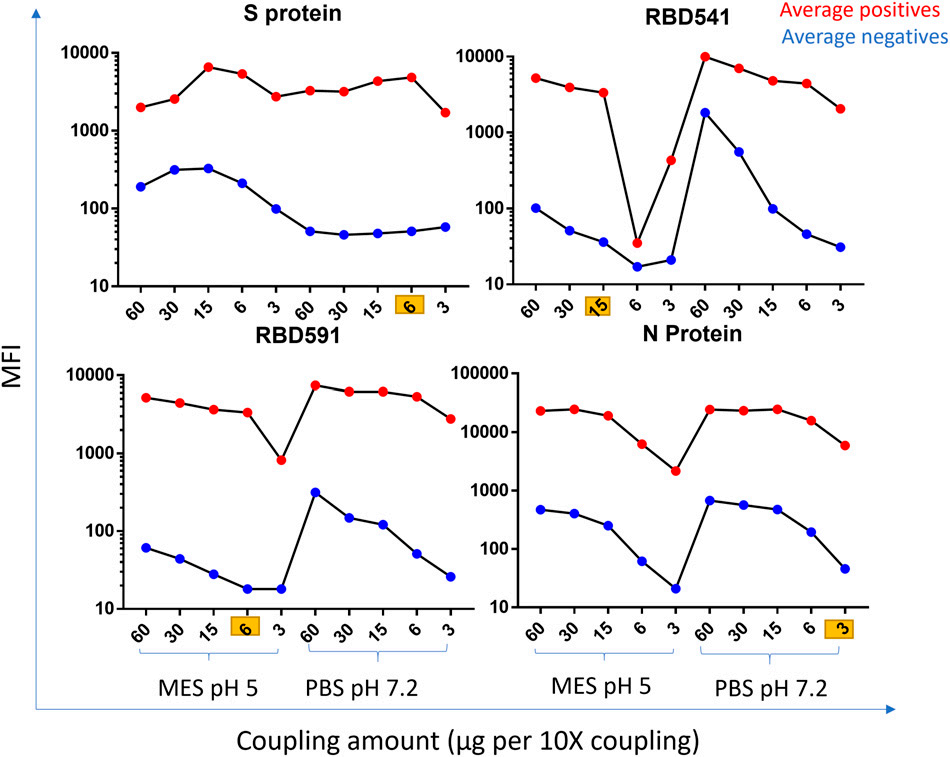
Coupling optimization on MAGPIX. MFI by coupling amount (µg per 10× coupling) and coupling buffer for each antigen run on MAGPIX. The average MFI of positive specimens is shown in red, and the average MFI of negative specimens is shown in blue. MFI = median fluorescence intensity; MES = 2-ethanesulfonic acid; MES pH 5 = 50 mM MES, 0.85% NaCl at pH 5; PBS pH 7.2 = 1x phosphate buffered saline pH 7.2; S = spike; RBD = receptor binding domain; N = nucleocapsid.

The optimal concentration of antigen per 12.5 × 10^6^ beads was 6 µg for S protein, 15 µg for RBD541, 6 µg for RBD591, and 3 µg for N protein. The optimal coupling buffer pH was 7.2 for S and N protein and 5 for RBD541, and RBD591. Average MFI values for positive and negative specimens on MAGPIX and Bio-Plex are shown in Figure 1 and Supplemental Figure 1 for each coupling condition.

**Figure 2.**
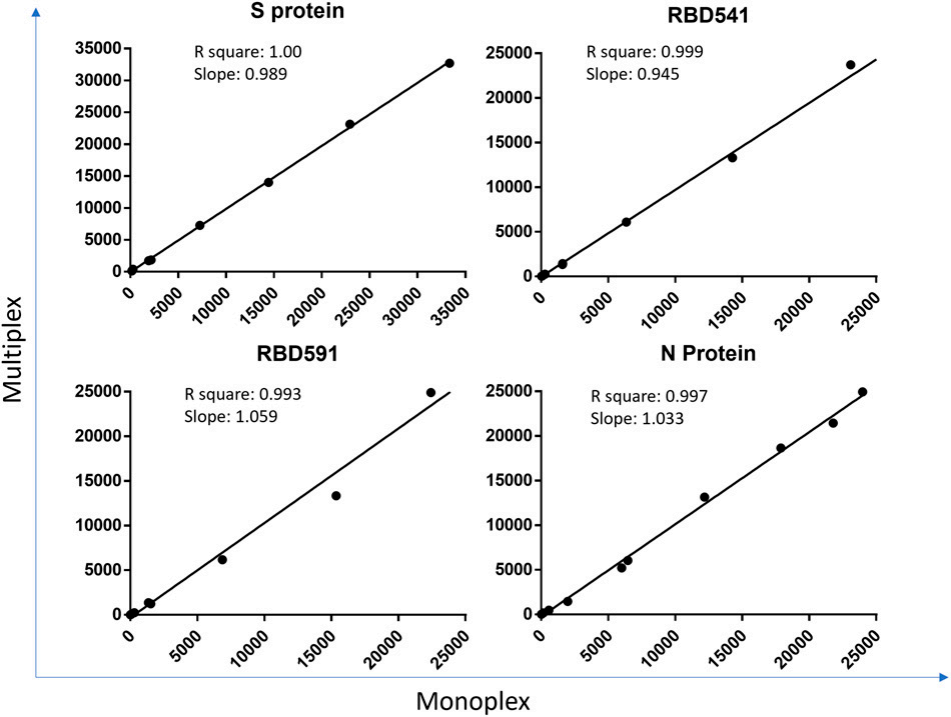
Comparison of monoplex and multiplex on MAGPIX. Coefficient of determination (R square) and slope between MFI-bg values of specimens tested in multiplex (all 4 antigens) and monoplex (each antigen individually) on MAGPIX are shown for each antigen. Each dot is an individual specimen. S = spike; RBD = receptor binding domain; N = nucleocapsid; MFI-bg = median fluorescence intensity minus background. This figure appears in color at www.ajtmh.org.

**Figure 3.**
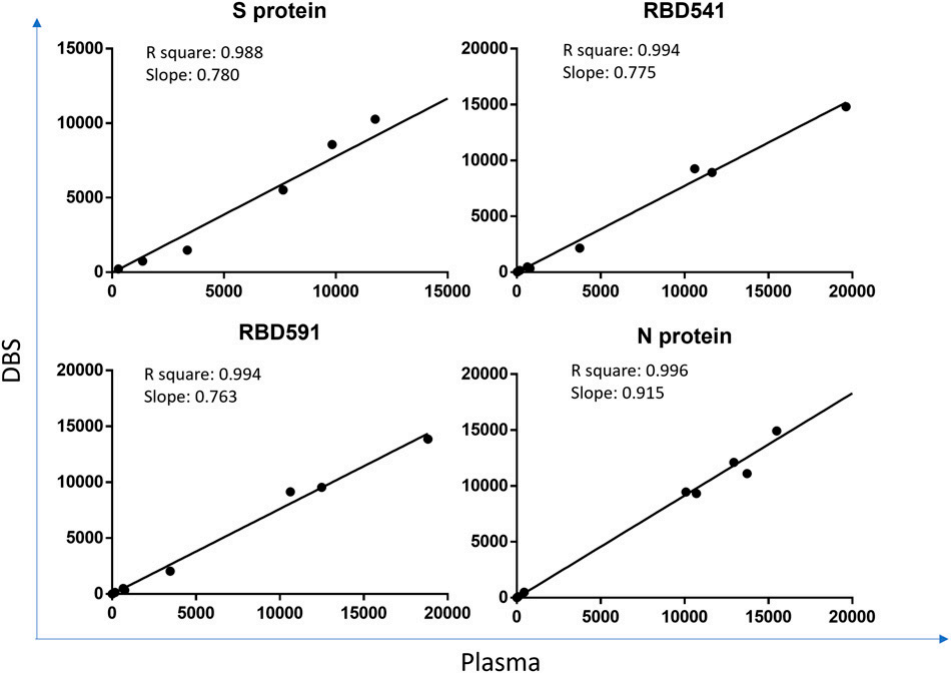
Comparison of plasma and DBS on MAGPIX. Coefficient of determination (R square) and slope between MFI-bg values of plasma and dried blood spots (DBS) on MAGPIX are shown for each antigen. Each dot is an individual specimen. DBS = dried blood spot; S = spike; RBD = receptor binding domain; N = nucleocapsid; MFI-bg = median fluorescence intensity minus background. This figure appears in color at www.ajtmh.org.

The correlation coefficient for each antigen on MAGPIX was > 0.99 between monoplex and multiplex and > 0.988 between plasma and DBS (Figures 2 and 3). Bio-Plex results are shown in Supplemental Figures 2 and 3.

### Cutoff values, sensitivity, and specificity.

**Figure 4.**
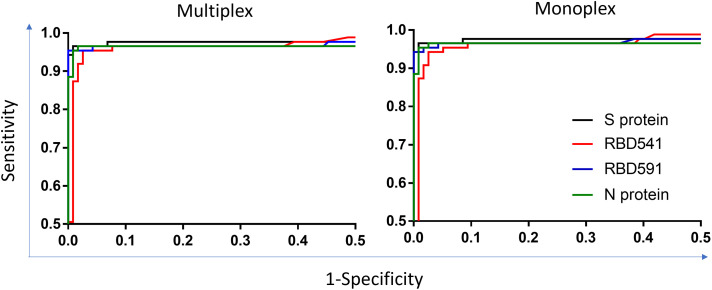
ROC plots for monoplex and multiplex testing on MAGPIX. Area under the curve values with 95% confidence intervals in parenthesis on each graph. ROC = receiver operating characteristic; S = spike; RBD = receptor binding domain; N = nucleocapsid.

The cutoff for positivity on MAGPIX was an MFI-bg value of 130 for S protein, 665 for RBD541, 627 for RBD591, and 1046 for N protein. The cutoff for positivity on Bio-Plex was an MFI-bg value of 1288 for S protein, 2919 for RBD541, 2653 for RBD591, and 3683 for N protein. Table 2 and Supplemental Table 1 show the sensitivity/specificity for each antigen tested in monoplex and multiplex on MAGPIX and Bio-Plex. Sensitivity ranged from 94.3% to 96.6% and specificity ranged from 97.4% to 100%. There were no differences in sensitivity or specificity when specimens were tested in monoplex or multiplex. Receiver operating characteristic curves for monoplex and multiplex testing on MAGPIX and Bio-Plex are shown in Figure 4 and Supplemental Figure 4.

**Table 2:** Sensitivity and specificity for monoplex and multiplex testing on MAGPIX

		Multiplex (95% CI)	Monoplex (95% CI)
S protein	Sensitivity	96.6% (90.3–99.3)	96.6% (90.3–99.3)
	Specificity	99.2% (95.3–100)	99.2% (93.4–100)
RBD541	Sensitivity	95.4% (88.6–98.7)	94.3% (87.1–98.7)
	Specificity	97.4% (92.7–99.5)	97.4% (92.7–99.5)
RBD591	Sensitivity	95.4% (88.6–98.7)	94.3% (87.1–98.1)
	Specificity	100% (96.9–100)	100% (96.9–100)
N protein	Sensitivity	96.5% (90.3–99.3)	95.4% (88.6–98.7)
	Specificity	98.3% (94.0–99.8)	99.2% (95.3–100)

S = spike; RBD = receptor binding domain; N = nucleocapsid. 95% confidence interval (CI) shown in parentheses.

**Table 3:** PPA and NPA for each antigen on MAGPIX

	PPA			NPA
	Overall (*N* = 108)	≤ 21 days (*N* = 54)	> 21 days (*N* = 48)	Overall (*N* = 97)
S protein	87.9% (80–92.8)	81.5% (69.2–89.6)	97.9% (89.1–100)	100% (96.2–100)
RBD541	76.9% (68.1–83.8)	63.0% (49.6–74.5)	93.8% (83.2–97.9)	99.0% (94.4–100)
RBD591	75.9% (68.0–83.8)	61.1% (47.8–73.0)	93.8% (83.2–97.9)	99.0% (94.4–100)
N protein	75.0% (66.1–82.2)	55.6% (42.4–68.0)	97.9% (89.1–99.9)	100% (96.2–100)

N = nucleocapsid; NPA = negative percent agreement; PPA = positive percent agreement; RBD = receptor binding domain; S = spike. PPA by ≤ 21 days and >21 days post-symptom onset is shown for each antigen. 95% confidence intervals are shown in parenthesis.

### Assay evaluation.

**Figure 5.**
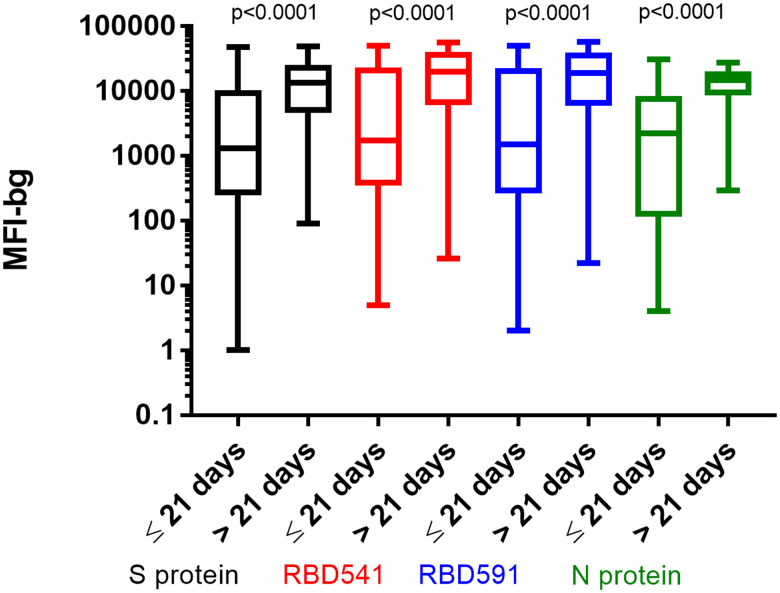
MFI-bg by days post symptom onset for each antigen on MAGPIX. Box and whiskers plots of MFI-bg values for S protein in black, RBD541 in red, RBD591 in blue and N protein in green. *P* values between ≤ 21 days and > 21 days post symptom onset for each antigen shown at the top of the graph. S = spike; RBD = receptor binding domain; N = nucleocapsid; MFI-bg = median fluorescence intensity minus background.

Positive percent agreement stratified by days since symptom onset and NPA for specimens tested at U.S. CDC on MAGPIX and Bio-Plex are shown in Table 3 and Supplemental Table 2. For all antigens, PPA was higher > 21 days post-symptom onset (93.8–97.9%) compared with ≤ 21 days post-symptom onset (55.6–81.5%). Median fluorescence intensity-bg values on MAGPIX and Bio-Plex were significantly higher in patients who were more than 21 days post-symptom onset for all antigens (Figure 5, and Supplemental Figure 5). Negative percent agreement was ≥ 99% for all antigens. We found no significant improvement in PPA/NPA using any combination of the four antigens compared with using the S protein alone (data not shown).

### Interlaboratory reproducibility.

Repeatability and reproducibility for each antigen and specimens on MAGPIX are shown in Table 4. Percent CVs for repeatability ranged from 0.5% to 11% and reproducibility ranged from 2.7% to 21.6%. Bio-Plex data are shown in Supplemental Table 3.

**Table 4:** Repeatability and reproducibility testing on MAGPIX

Antigen	MFI-bg	Sample	Repeatability (% CV)	Reproducibility (% CV)
S protein	19,102	P1	2.2	3.5
	8,338	P2	3.9	11.3
	2,772	P3	5.8	14.6
	1,135	P4	4.5	14.6
	1,240	P5	5.1	14.7
	41,409	P6	1.5	4.6
RBD_541_	13,594	P1	1.8	9.7
	9,541	P2	4.9	10.6
	3,322	P3	11.0	14.2
	1,322	P4	7.1	9.3
	2,381	P5	3.9	16.8
	43,631	P6	0.7	2.8
RBD_591_	12,748	P1	2.0	10.5
	10,136	P2	4.4	11.5
	3,649	P3	10.7	17.2
	1,450	P4	6.2	8.4
	2,268	P5	3.3	18.0
	44,442	P6	0.5	2.7
N protein	21,033	P1	2.9	5.2
	10,957	P2	7.0	13.8
	6,614	P3	3.5	14.0
	3,688	P4	4.6	21.6
	10,355	P5	4.8	16.9
	28,203	P6	3.4	6.9

CV = coefficient of variation; MFI-bg = median fluorescence intensity minus background; N = nucleocapsid; P1–P6 = positive specimen 1–positive specimen 6; RBD = receptor binding domain; S = spike. The average MFI-bg for each specimen run on multiple days is shown for each antigen. Percent CV is shown for each antigen and specimen for repeatability testing (multiple plates, one operator) and reproducibility testing (multiple plates, multiple operators, two laboratories).

**Figure 6.**
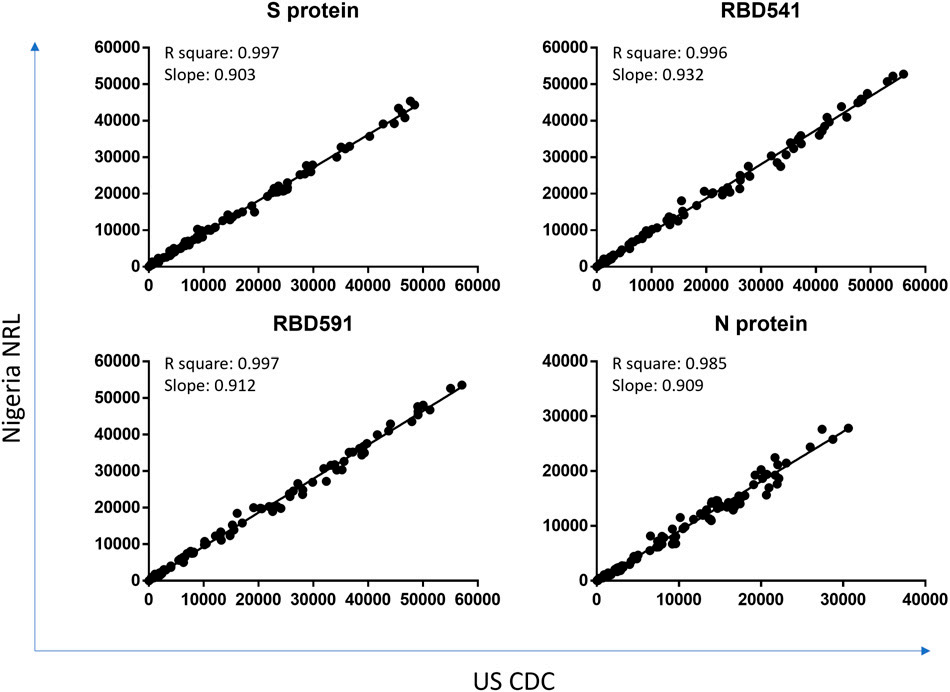
Comparison of testing at CDC and NRL on MAGPIX. Coefficient of determination (R square) and slope between MFI-bg values of specimens run at US CDC and Nigeria NRL on MAGPIX are shown for each antigen. S = spike; RBD = receptor binding domain; N = nucleocapsid; MFI-bg = median fluorescence intensity minus background; US CDC = United States Centers for Disease Control and Prevention; NRL = National Reference Laboratory. This figure appears in color at www.ajtmh.org.

The R^2^ for each antigen between testing at U.S. CDC and Nigeria NRL was 0.985 or above (Figure 6). The overall percent agreement in specimens classified as positive or negative in each location was 100% for S protein, 99.2% for RBD541, 98.3% for RBD591, and 97.5% for N protein.

## DISCUSSION

We show here that these four SARS-CoV-2 antigens show good performance when used in our existing MBA with high sensitivity, specificity, PPA, and NPA. The test exceeded the United States Food and Drug Administration serology requirements of 90% sensitivity and 95% specificity based on an ROC analysis.^30^ Positive percent agreement on an additional set of rRT-PCR positive specimens was higher for individuals with specimens collected > 21 days post-symptom onset than those collected ≤ 21 days post-symptom onset, similar to other SARS-CoV-2 serology assays.^30^ The latter individuals likely had yet to develop detectable antibody responses.^31^ The test performance was excellent based on the ROC area under the curve, with high precision and inter-laboratory reproducibility. Integrated serological testing is increasingly being used in laboratories worldwide, and data here support that bead-based multiplex assays provide robust platforms for detection of antibodies to SARS-CoV-2.

This assay adds to the growing number of multiplexed assays that can simultaneously test for antibodies against multiple SARS-CoV-2 antigens with routinely high sensitivity and specificity^13^^,^^32^^–^^36^ and high repeatability.^37^ While increased sensitivity was achieved using a multi-antigen for many assays,^13^^,^^32^^–^^35^^,^^37^ we found no significant improvement in PPA or NPA using any combination of the four antigens compared with using only the S protein alone. We will continue evaluate in other populations, particularly in those with malaria exposure, for which cross-reactivity with SARS-CoV-2 antigens has been documented.^14^ However, the ability to achieve high sensitivity with a single antigen would facilitate using antibodies to N and S antigens to potentially differentiate vaccine-induced versus infection-induced immunity for S only based vaccines. Bead-based immunoassays tend to have high analytical sensitivity (i.e., can detect small amounts of antibody in a specimen) from small specimen volumes; our assay uses a 1:400 dilution of serum. Multiple readings—50 in this assay—are taken for each antigen in a single well to obtain a robust measure of MFI. Excellent test performance was seen for two different instruments, the MAGPIX and Luminex-200, giving flexibility to laboratories interested in multiplex-based testing. The high interlaboratory agreement testifies to the ruggedness of the assay to withstand variations in external parameters, such as shipping specimens, different instruments in different facilities, and operator variation (of note, NRL uses an assembly line approach to MBA testing so nine individuals had a role in running all 12 plates). The robustness of the MBA, coupled with the ability to simultaneously test for antibodies to multiple antigens, make this an appealing option for large-scale serosurveys.

While serology tests as a whole tend to lack well-defined controls to assess sensitivity and specificity, SARS-CoV-2 serology tests have been developed under unusual circumstances: the worldwide focus on this pandemic has resulted in collection of unusually well-defined specimen panels to determine test performance. Among these are specimens for which the time from infection or symptom onset is documented, allowing good estimates of the time frame after infection in which antibodies in blood will be detectable by available tests. By contrast, we did not include antigens from seasonal coronaviruses due to the lack of well-defined panels from people with laboratory-confirmed cases of other coronaviruses. The assay will also further be evaluated as new variants emerge.

The COVID-19 pandemic has led to a wider understanding of the utility of serosurveys in understanding population-level exposure to infectious agents. While serology is not currently part of WHO guidance for diseases such as malaria or NTDs with elimination targets, serology is currently part of WHO guidance and verification of elimination for VPDs.^38^ Additionally, serology is becoming increasingly used to estimate prevalence to help programs prioritize where to focus interventions.^39^^,^^40^ Multiplex testing of DBS collected during a serosurvey triggered by a diphtheria outbreak in refugee camps in Cox’s Bazar, Bangladesh, showed little evidence of malaria,^22^ trachoma,^17^ or yaws.^17^ An NTD collateral benefits study provided critical data to show tetanus immunity gaps in East African adult men.^25^ Unknown pockets of *Strongyloides stercoralis* infection in Cambodia were revealed by multiplex testing of specimens from a VPD serosurvey.^18^ While this study only used SARS-CoV-2 antigens due to the restrictions of the consent for testing, we have not observed any decreased performance when adding other antigens to this assay so do not expect that to be a problem. The ability to test for antibodies to SARS-CoV-2 at the same time as over two dozen other infectious agents in the MBA creates an opportunity for COVID-19 serosurveys to be vehicles to gain more transmission data on other diseases of public health interest, and further data on SARS-CoV-2 transmission could be obtained through serosurveys for other diseases.

The 17th and final goal of the United Nation Sustainable Development Goals is “Partnerships for the Goals.”^41^ The concept of partnership is further delineated in the 2021–2030 NTD Road Map,^42^ which has as one of its three pillars “holistic, cross-cutting approaches including integration across NTDs, mainstreaming in national health systems, coordinating with adjacent sectors and strengthening country capacity and global support.” While serosurvey data will not necessarily overcome the delays to program implementation imposed by the COVID-19 pandemic,^43^^–^^45^ early adoption of integrated approaches will have long-term benefits for a variety of disease programs by establishing strong partnership to achieve sustainable development goals.

## Supplemental Material


Supplemental materials

## References

[1] Wolfel R 2020. Virological assessment of hospitalized patients with COVID-2019. Nature 581: 465–469. 32235945 10.1038/s41586-020-2196-x

[2] Qu J Wu C Li X Zhang G Jiang Z Li X Zhu Q Liu L , 2020. Profile of immunoglobulin G and IgM antibodies against severe acute respiratory syndrome coronavirus 2 (SARS-CoV-2). Clin Infect Dis 71: 2255–2258. 32337590 10.1093/cid/ciaa489 PMC7197626

[3] Bajema KL 2021. Estimated SARS-CoV-2 seroprevalence in the US as of September 2020. JAMA Intern Med 181: 450–460. 33231628 10.1001/jamainternmed.2020.7976 PMC7686880

[4] Basavaraju SV 2021. Serologic testing of US blood donations to identify severe acute respiratory syndrome coronavirus 2 (SARS-CoV-2)-reactive antibodies: December 2019–January 2020. Clin Infect Dis 72: e1004–e1009. 33252659 10.1093/cid/ciaa1785 PMC7799215

[5] Pathela P 2021. Seroprevalence of SARS-CoV-2 following the largest initial epidemic wave in the United States: findings from New York City, May 13–July 21, 2020. *J Infect Dis 224:* 196–206. 10.1093/infdis/jiab200 PMC8083309 33836067

[6] Reyes-Vega MF Soto-Cabezas MG Cardenas F Martel KS Valle A Valverde J Vidal-Anzardo M Falcon ME Munayco CV Peru C-WG , 2021. SARS-CoV-2 prevalence associated to low socioeconomic status and overcrowding in an LMIC megacity: a population-based seroepidemiological survey in Lima, Peru. EClinicalMedicine 34: 100801. 33817611 10.1016/j.eclinm.2021.100801 PMC8009628

[7] Etyang AO 2022. Seroprevalence of antibodies to SARS-CoV-2 among health care workers in Kenya. Clin Infect Dis.74: 288–293 10.1093/cid/ciab346 PMC8135298 33893491

[8] Uyoga S 2021. Seroprevalence of anti-SARS-CoV-2 IgG antibodies in Kenyan blood donors. Science 371: 79–82. 33177105 10.1126/science.abe1916 PMC7877494

[9] NCDC and NIMR Release Findings of COVID-19 Household Seroprevalence Surveys in Four States of Nigeria Available at: https://reliefweb.int/report/nigeria/ncdc-and-nimr-release-findings-covid-19-household-seroprevalence-surveys-four-states.

[10] Al Suwaidi H Senok A Varghese R Deesi Z Khansaheb H Pokasirakath S Chacko B Abufara I Loney T Alsheikh-Ali A , 2021. Saliva for molecular detection of SARS-CoV-2 in school-age children. Clin Microbiol Infect. 13: 3303–3399. 10.1016/j.cmi.2021.02.009 PMC7894096 33618013

[11] Poljak M Ostrbenk Valencak A Stamol T Seme K , 2021. Head-to-head comparison of two rapid high-throughput automated electrochemiluminescence immunoassays targeting total antibodies to the SARS-CoV-2 nucleoprotein and spike protein receptor binding domain. J Clin Virol 137: 104784. 33711693 10.1016/j.jcv.2021.104784 PMC7934695

[12] Chen X 2021. Serological evidence of human infection with SARS-CoV-2: a systematic review and meta-analysis. Lancet Glob Health 9: e598–e609. 33705690 10.1016/S2214-109X(21)00026-7 PMC8049592

[13] Fotis C 2021. Accurate SARS-CoV-2 seroprevalence surveys require robust multi-antigen assays. Sci Rep 11: 6614. 33758278 10.1038/s41598-021-86035-2 PMC7988055

[14] Steinhardt LC 2021. Cross-reactivity of two SARS-CoV-2 serological assays in a setting where malaria is endemic. J Clin Microbiol 59: e0051421. 33853839 10.1128/JCM.00514-21 PMC8218747

[15] Arnold BF Scobie HM Priest JW Lammie PJ , 2018. Integrated serologic surveillance of population immunity and disease transmission. Emerg Infect Dis 24: 1188–1194. 29912680 10.3201/eid2407.171928 PMC6038749

[16] Lammie PJ Moss DM Brook Goodhew E Hamlin K Krolewiecki A West SK Priest JW , 2012. Development of a new platform for neglected tropical disease surveillance. Int J Parasitol 42: 797–800. 22846784 10.1016/j.ijpara.2012.07.002

[17] Cooley GM 2021. No serological evidence of trachoma or yaws among residents of registered camps and makeshift settlements in Cox’s Bazar, Bangladesh. Am J Trop Med Hyg. 104: 2031–2037 10.4269/ajtmh.21-0124 PMC8176462 33939630

[18] Priest JW 2016. Integration of multiplex bead assays for parasitic diseases into a national, population-based serosurvey of women 15–39 years of age in Cambodia. PLoS Negl Trop Dis 10: e0004699. 27136913 10.1371/journal.pntd.0004699 PMC4854427

[19] Priest JW Moss DM Arnold BF Hamlin K Jones CC Lammie PJ , 2015. Seroepidemiology of toxoplasma in a coastal region of Haiti: multiplex bead assay detection of immunoglobulin G antibodies that recognize the SAG2A antigen. Epidemiol Infect 143: 618–630. 25600668 10.1017/S0950268814001216 PMC5844480

[20] Corran P Coleman P Riley E Drakeley C , 2007. Serology: a robust indicator of malaria transmission intensity? Trends Parasitol 23: 575–582. 17988945 10.1016/j.pt.2007.08.023

[21] Drakeley CJ 2005. Estimating medium- and long-term trends in malaria transmission by using serological markers of malaria exposure. Proc Natl Acad Sci USA 102: 5108–5113. 15792998 10.1073/pnas.0408725102 PMC555970

[22] Lu A 2020. Screening for malaria antigen and anti-malarial IgG antibody in forcibly-displaced Myanmar nationals: Cox’s Bazar district, Bangladesh, 2018. Malar J 19: 130. 32228699 10.1186/s12936-020-03199-4 PMC7106647

[23] Moss DM Priest JW Hamlin K Derado G Herbein J Petri WA Jr Lammie PJ , 2014. Longitudinal evaluation of enteric protozoa in Haitian children by stool exam and multiplex serologic assay. Am J Trop Med Hyg 90: 653–660. 24591430 10.4269/ajtmh.13-0545 PMC3973509

[24] Priest JW Moss DM , 2020. Measuring cryptosporidium serologic responses by multiplex bead assay. Methods Mol Biol 2052: 61–85. 31452157 10.1007/978-1-4939-9748-0_5

[25] Scobie HM 2017. Tetanus immunity gaps in children 5-14 years and men ≥ 15 years of age revealed by integrated disease serosurveillance in Kenya, Tanzania, and Mozambique. Am J Trop Med Hyg 96: 415–420. 27920395 10.4269/ajtmh.16-0452 PMC5303047

[26] Gwyn S Mitchell A Dean D Mkocha H Handali S Martin DL , 2016. Lateral flow-based antibody testing for *Chlamydia trachomatis.* J Immunol Methods 435: 27–31. 27208400 10.1016/j.jim.2016.05.008

[27] Kainulainen MH 2021. High-throughput quantitation of SARS-CoV-2 antibodies in a single-dilution homogeneous assay. Sci Rep 11: 12330. 34112850 10.1038/s41598-021-91300-5 PMC8192771

[28] Hsieh CL 2020. Structure-based design of prefusion-stabilized SARS-CoV-2 spikes. Science 369: 1501–1505. 32703906 10.1126/science.abd0826 PMC7402631

[29] FDA , 2021. *Serology Template for Test Developers*. Available at: https://www.fda.gov/medical-devices/coronavirus-disease-2019-covid-19-emergency-use-authorizations-medical-devices/in-vitro-diagnostics-euas.

[30] U.S. Food and Drug Administration *FDA Serology Template for Test Developers*. Available at: https://www.fda.gov/media/137698/download.

[31] Dan JM 2020. Immunological memory to SARS-CoV-2 assessed for up to eight months after infection. Science 371: eabf4063. 10.1126/science.abf4063 PMC7919858 33408181

[32] Becker M 2021. Exploring beyond clinical routine SARS-CoV-2 serology using MultiCoV-Ab to evaluate endemic coronavirus cross-reactivity. Nat Commun 12: 1152. 33608538 10.1038/s41467-021-20973-3 PMC7896075

[33] Butt J 2021. From multiplex serology to serolomics-a novel approach to the antibody response against the SARS-CoV-2 proteome. *Viruses* 13: 749. 33923338 10.3390/v13050749 PMC8147094

[34] Favresse J Brauner J Bodart N Vigneron A Roisin S Melchionda S Douxfils J Ocmant A , 2021. An original multiplex method to assess five different SARS-CoV-2 antibodies. Clin Chem Lab Med 59: 971–978. 33554567 10.1515/cclm-2020-1652

[35] Rosado J 2021. Multiplex assays for the identification of serological signatures of SARS-CoV-2 infection: an antibody-based diagnostic and machine learning study. Lancet Microbe 2: e60–e69. 33521709 10.1016/S2666-5247(20)30197-X PMC7837364

[36] Schultz JS 2021. Development and validation of a multiplex microsphere immunoassay using dried blood spots for SARS-CoV-2 seroprevalence: application in first responders in Colorado, USA. *J Clin Microbiol* 59: e00290–21. 33795412 10.1128/JCM.00290-21 PMC8315929

[37] Hartog N Faber W Frisch A Bauss J Bupp CP Rajasekaran S Prokop JW , 2021. SARS-CoV-2 infection: molecular mechanisms of severe outcomes to suggest therapeutics. Expert Rev Proteomics 18: 105–118. 33779460 10.1080/14789450.2021.1908894 PMC8022340

[38] World Health Organization , 2020. *Measles and Rubella Strategic Framework 2021–2030*.

[39] Moss WJ et al., 2021. Feasibility assessment of measles and rubella eradication. Vaccine 39: 3544–3559. 34045102 10.1016/j.vaccine.2021.04.027

[40] Patel MK 2020. Progress toward regional measles elimination—worldwide, 2000–2019. MMWR Morb Mortal Wkly Rep 69: 1700–1705. 33180759 10.15585/mmwr.mm6945a6 PMC7660667

[41] Assembly UNG , 2015. Resolution of the General Assembly on 25 September 2015 (A/70/L.1). *Transforming Our World: The 2030 Agenda for Sustainable Development.* New York, NY: United Nations.

[42] WHO , 2021. Ending the Neglect to Attain the Sustainable Development Goals: A Framework for Monitoring and Evaluating Progress of the Road Map for Neglected Tropical Diseases 2021–2030. Geneva, Switzerland: World Health Organization.

[43] Hamley JID Blok DJ Walker M Milton P Hopkins AD Hamill LC Downs P de Vlas SJ Stolk WA Basanez MG , 2021. What does the COVID-19 pandemic mean for the next decade of onchocerciasis control and elimination? Trans R Soc Trop Med Hyg 115: 269–280. 33515042 10.1093/trstmh/traa193 PMC7928565

[44] Prada JM 2021. Delays in lymphatic filariasis elimination programmes due to COVID-19, and possible mitigation strategies. Trans R Soc Trop Med Hyg 115: 261–268. 33515454 10.1093/trstmh/trab004 PMC7928650

[45] Toor J 2021. Predicted impact of COVID-19 on neglected tropical disease programs and the opportunity for innovation. Clin Infect Dis 72: 1463–1466. 32984870 10.1093/cid/ciaa933 PMC7543306

