# Supplementary material for: Performance of SARS-CoV-2 Antigens in a Multiplex Bead Assay for Integrated Serological Surveillance of Neglected Tropical and Other Diseases
Source: Am J Trop Med Hyg. 2022 Jun 27;107(2):260–7. doi: 10.4269/ajtmh.22-0078 (PMC9393470; doi:10.4269/ajtmh.22-0078)
Supplement: Supplementary file 1 [file tpmd220078.SD1.pdf]

Supplemental Figures

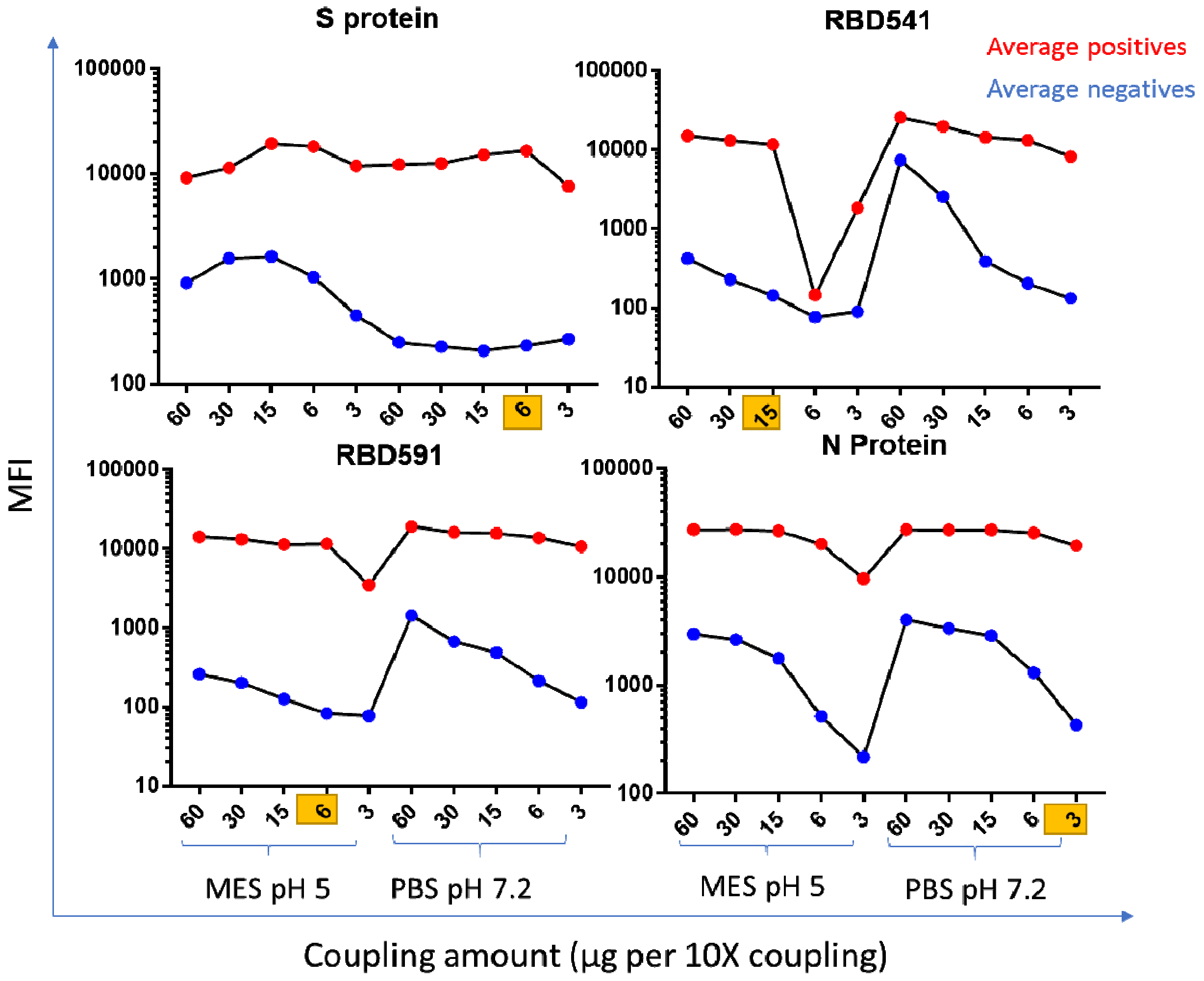

Supplemental Figure S1: Coupling optimization on Bio-Plex. MFI by coupling amount (µg per 10X coupling) and coupling buffer for each antigen run on Bio-Plex. The average MFI of positive specimens is shown in red, and the average MFI of negative specimens is shown in blue. MFI = median fluorescence intensity; MES = 2-ethanesulfonic acid; MES pH 5 = 50 mM MES, 0.85% NaCl at pH 5; PBS pH 7.2 = 1X phosphate buffered saline pH 7.2; S = spike; RBD = receptor binding domain; N = nucleocapsid.

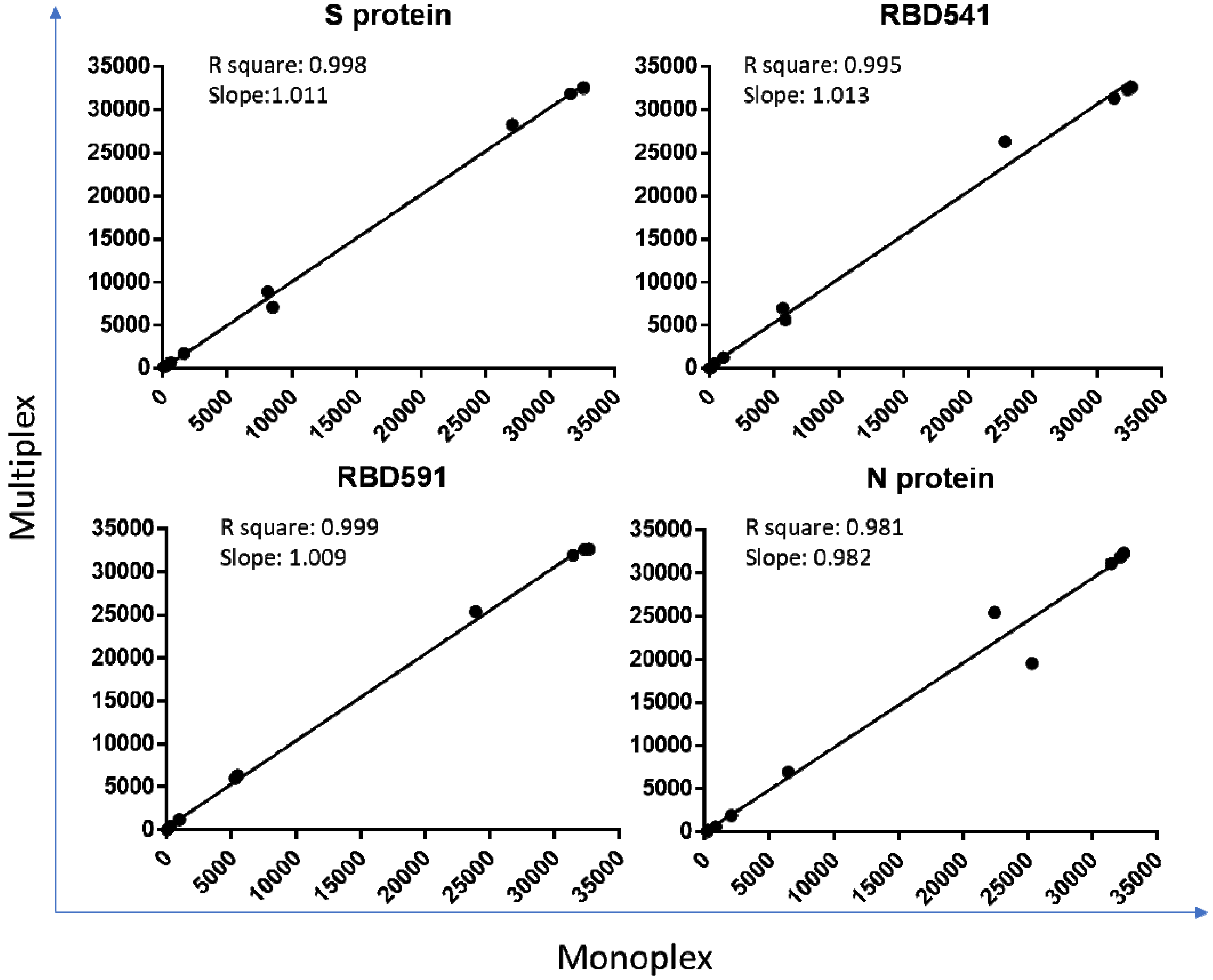

Supplemental Figure S2: Comparison of monoplex and multiplex on Bio-Plex. Coefficient of determination (R square) and slope between MFI-bg values of specimens tested in multiplex (all 4 antigens) and monoplex (each antigen individually) on Bio-Plex are shown for each antigen. Each dot is an individual specimen. S = spike; RBD = receptor binding domain; N = nucleocapsid; MFI-bg = median fluorescence intensity minus background

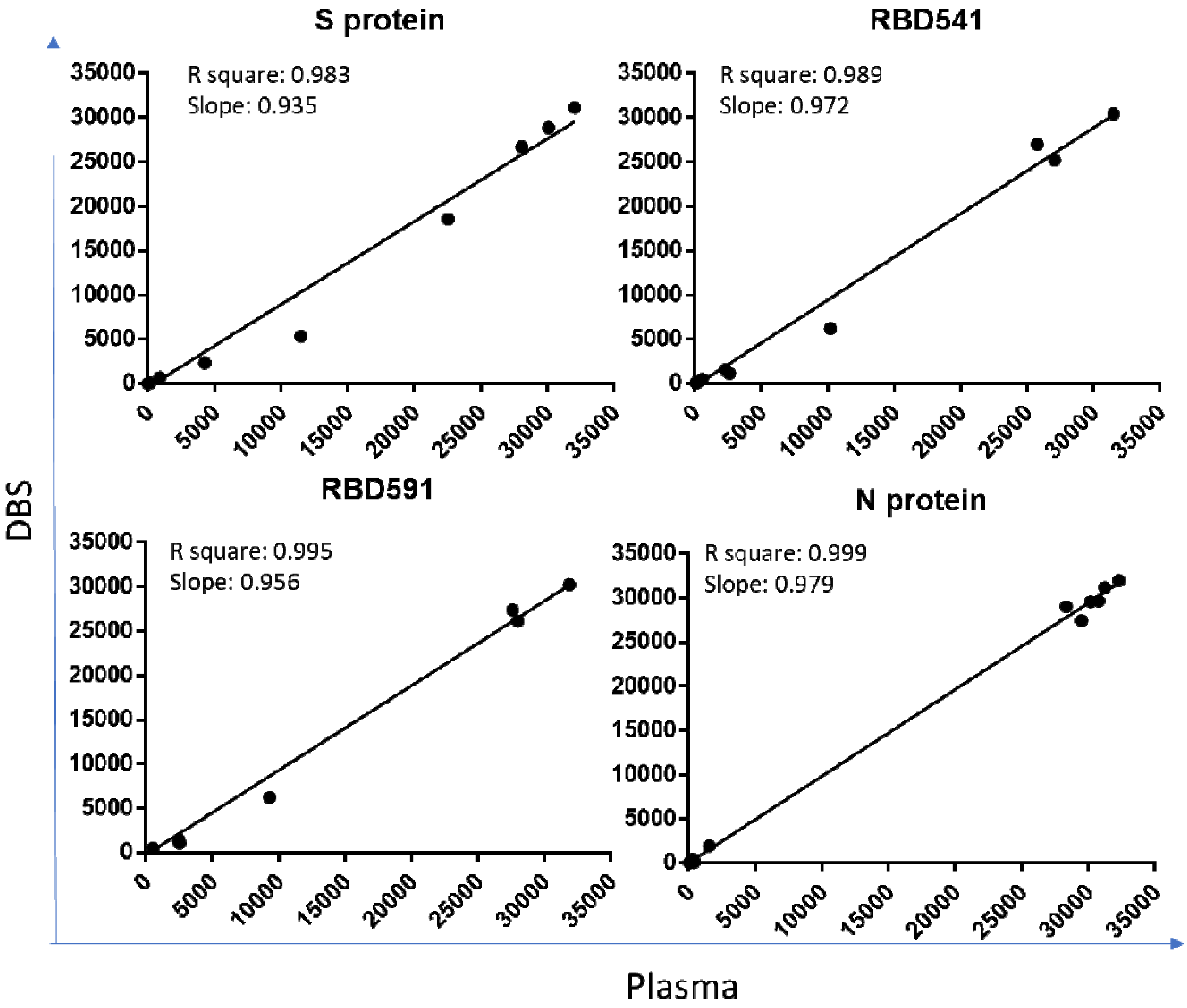

Supplemental Figure S3: Comparison of plasma and DBS on Bio-Plex. Coefficient of determination (R square) and slope between MFI-bg values of plasma and dried blood spots (DBS) on Bio-Plex are shown for each antigen. Each dot is an individual specimen. DBS = dried blood spot; S = spike; RBD = receptor binding domain; N = nucleocapsid; MFI-bg = median fluorescence intensity minus background

		Multiplex (95% CI)	Monoplex (95% CI)
**S protein**	**Sensitivity**	95.4% (88.6-98.7)	96.5% (90.3-99.3)
	**Specificity**	99.2% (95.3-1.00)	99.2% (95.3-1.00)
**RBD541**	**Sensitivity**	95.4% (88.6-98.7)	95.4% (88.6-98.7)
	**Specificity**	97.4% (92.7-99.5)	96.6% (91.5-99.1)
**RBD591**	**Sensitivity**	95.4% (88.6-98.7)	95.4% (88.6-98.7)
	**Specificity**	100% (96.9-100)	99.2% (95.3-100)
**N protein**	**Sensitivity**	96.5% (90.3-99.3)	96.6% (90.3-99.3)
	**Specificity**	97.4% (90.3-99.3)	98.3% (93.4-100)

Supplemental Table S1: Sensitivity and specificity for monoplex and multiplex testing on Bio-Plex. 95% confidence interval (CI) shown in parentheses. S = spike; RBD = receptor binding domain; N = nucleocapsid.

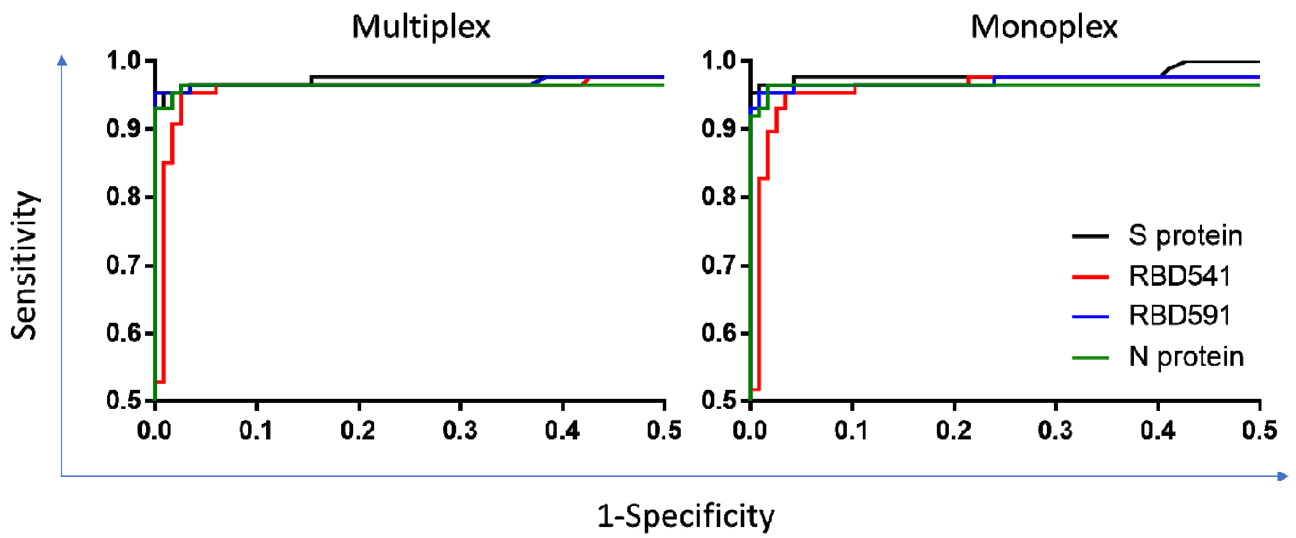

Supplemental Figure S4: ROC plots for monoplex and multiplex testing on Bio-Plex. Area under the curve values with 95% confidence intervals for multiplex and monoplex testing, respectively, for each antigen are 0.980 (0.954-1.005) and 0.999 (0.977-1.003) for S protein, 0.973 (0.946-1.000) and 0.975 (0.951-0.999) for RBD541, 0.976 (0.947-1.004) and 0.976 (0.946-1.005) for RBD591, and 0.968 (0.933-1.003) and 0.968 (0.932-1.003) for N protein. ROC = receiver operating characteristic; S = spike; RBD = receptor binding domain; N = nucleocapsid.

	PPA			NPA
	**Overall (n=108)**	**≤ 21 days (n=54)**	**> 21 days (n=48)**	**Overall (n=97)**
**S protein**	83.3% (75.2-89.2)	72.2% (59.1-82.4)	97.9% (89.1-99.9)	100% (96.2-100)
**RBD541**	75.9% (67.1-83.0)	61.1% (47.8-73.0)	93.8% (83.2-97.9)	100% (96.2-100)
**RBD591**	75.0% (66.1-82.2)	59.3% (46.0-71.3)	93.8% (83.2-97.9)	99.0% (94.4-100)
**N protein**	74.0% (65.0-81.4)	53.7% (66.3-40.6)	97.9% (89.1-99.9)	100% (96.2-100)

Supplemental Table S2: PPA and NPA for each antigen on Bio-Plex. PPA by ≤ 21 days and >21 days post symptom onset is shown for each antigen. 95% confidence intervals are shown in parenthesis. S = spike; RBD = receptor binding domain; N = nucleocapsid; PPA = positive percent agreement; NPA = negative percent agreement

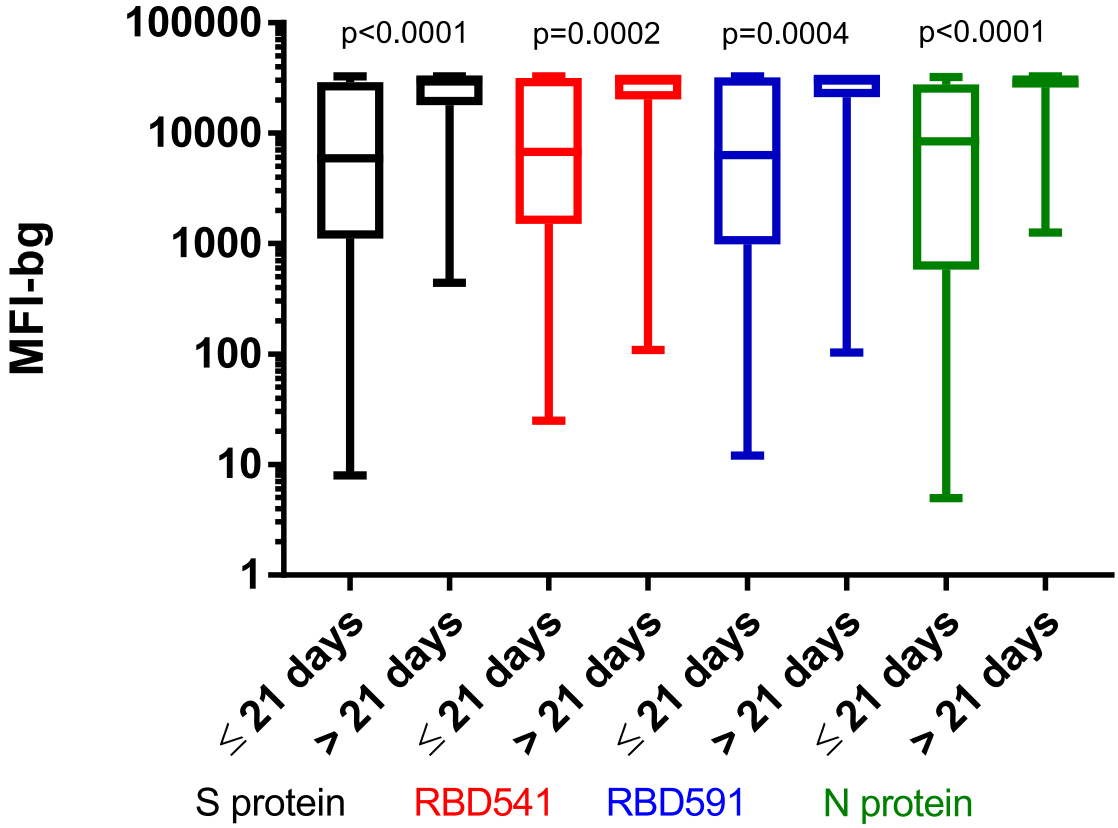

Supplemental Figure S5: MFI-bg by days post symptom onset for each antigen on Bio-Plex. Box and whiskers plots of MFI-bg values for S protein in black, RBD541 in red, RBD591 in blue and N protein in green. P values between ≤ 21 days and > 21 days post symptom onset for each antigen shown at the top of the graph. S = spike; RBD = receptor binding domain; N = nucleocapsid; MFI-bg = median fluorescence intensity minus background.

	MFI-bg	Sample	Repeatability (%CV)	Int. Precision (%CV)
**S protein**	29912	**P1**	3.5	3.5
	25334	**P2**	4.0	3.8
	11426	**P3**	10.7	11.7
	4673	**P4**	6.8	8.4
	5077	**P5**	13.1	12.3
	31826	**P6**	1.9	1.6
**RBD_{541}**	27772	**P1**	5.3	5.3
	27772	**P1**	5.3	5.3
	25299	**P2**	5.8	4.9
	11384	**P3**	13.0	12.5
	4481	**P4**	10.9	9.6
	8180	**P5**	15.0	16.1
	31842	**P6**	1.9	1.6
**RBD_{591}**	27701	**P1**	5.5	5.1
	25898	**P2**	5.2	4.7
	12444	**P3**	8.5	9.5
	4886	**P4**	7.0	7.9
	7969	**P5**	11.3	14.7
	31938	**P6**	1.7	1.7
**N protein**	30433	**P1**	3.0	2.8
	27711	**P2**	4.5	4.9
	23458	**P3**	2.8	4.8
	12821	**P4**	7.4	8.6
	27333	**P5**	5.2	5.5
	31109	**P6**	2.5	2.4

Supplemental Table S3: Repeatability and reproducibility testing on Bio-Plex. The average MFI-bg for each specimen run on multiple days is shown for each antigen. Percent CV is shown for each antigen and specimen for repeatability testing (multiple plates, one operator) and intermediate precision testing (multiple plates, multiple operators). Int = intermediate; S = spike; RBD = receptor binding domain; N = nucleocapsid; MFI-bg = median fluorescence intensity minus background; CV = coefficient of variation; P1-P6 = positive specimen 1 – positive specimen 6.
